# Long‐term outcomes after stent implantation in very small vessel coronary artery disease

**DOI:** 10.1002/clc.24000

**Published:** 2023-02-23

**Authors:** En‐Shao Liu, Tse‐Hsuan Yang, Ta‐Hsin Tai, Cheng‐Hung Chiang, Chin‐Chang Cheng, Wei‐Chun Huang, Guang‐Yuan Mar, Feng‐Yu Kuo

**Affiliations:** ^1^ Cardiovascular Medical Center Kaohsiung Veterans General Hospital Kaohsiung Taiwan; ^2^ Institute of Clinical Medicine National Yang Ming Chiao Tung University Taipei Taiwan; ^3^ Department of Cardiology Kaohsiung Municipal United Hospital Kaohsiung Taiwan; ^4^ Department of Critical Care Medicine Kaohsiung Veterans General Hospital Kaohsiung Taiwan; ^5^ School of Medicine National Yang Ming Chiao Tung University Taipei Taiwan; ^6^ Department of Physical Therapy Fooyin University Kaohsiung Taiwan; ^7^ Graduate Institute of Clinical Medicine Kaohsiung Medical University Kaohsiung Taiwan; ^8^ Department of Pharmacy and Master Program, College of Pharmacy and Health Care Tajen University Pingtung Taiwan

**Keywords:** chronic kidney disease, drug‐eluting stent, percutaneous Coronary intervention, very small vessel coronary artery disease

## Abstract

**Background:**

Percutaneous coronary interventions (PCI) in very small vessel lesions represent an intriguing aspect of coronary artery disease (CAD). Uncertainty still exists in stent implantation in very small caliber vessels. This study aimed to evaluate the long‐term outcomes of patients treated with 2.0‐mm drug‐eluting stent (DES).

**Method:**

This retrospective observational study included 134 patients undergoing PCI with 2.0‐mm zotarolimus DES from December 2016 to May 2020. The primary endpoint was major adverse cardiovascular events (MACE) at 2‐year follow‐up, which was composed of all‐cause mortality, target vessel myocardial infarction, and ischemia‐driven target lesion revascularization. Multiple logistic regression analysis was used to identify the independent predictors of MACE, and odds ratios (OR) and 95% confidence intervals (CI) were calculated.

**Result:**

The lesions were diffuse (mean length 20.9 ± 5.51 mm) and belong to type B2/C lesions (90.3%). On follow‐up, the MACE rate was 20.1% and mostly driven by late lumen loss demanding revascularization (11.9%). In multivariable analysis, chronic kidney disease (CKD) (OR: 4.291, 95% CI: 1.574−11.704, *p* = 0.004) and calcified lesions (OR: 3.688, 95% CI: 1.311−10.371, *p* = 0.013) were the independent predictors of subsequent cardiovascular events, whereas statin was associated with better outcomes (OR: 0.335, 95% CI: 0.119−0.949, *p* = 0.040).

**Conclusion:**

2.0‐mm DES is a feasible option for treating very small vessel CAD in complex lesions. Patients with CKD and calcified lesions carry the hazard of worse outcomes, and careful consideration should be taken before stenting in this high‐risk population.

## Graphical abstract

Long‐term outcomes and associated factors of 2.0 mm drug‐eluting stent implantation in very small vessel coronary artery disease.

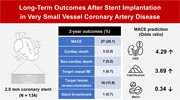

## 1 INTRODUCTION

Up to one‐third of patients undergoing percutaneous coronary intervention (PCI) have small vessel coronary artery disease (CAD), and it is especially prevalent in patients with diabetes mellitus (DM) and chronic kidney disease (CKD).^1^, ^2^, ^3^ PCI of small vessel lesions presents technical challenges and is associated with increased risks of late lumen loss and adverse cardiovascular events.^4^, ^5^, ^6^

The definition of small vessel CAD varies across trials until the Society for Cardiovascular Angiography recently published a literature suggesting a reference vessel diameter (RVD) < 2.5 mm as the cutoff value for classifying small coronary vessels.^5^, ^7^, ^8^ The evolution of stent technology and the availability of 2.25‐mm and 2.0‐mm drug‐eluting stents (DES) has led to very small vessel CAD being proposed to describe the lesions that are only amenable to these devices.^9^, ^10^ Although studies have indicated no significant difference in clinical outcome between 2.25 and 2.0‐mm zotarolimus DES at 1‐year follow‐up,^10^ the long‐term efficacy and safety of 2.0‐mm DES remain unclear. No consensus exists on an optimal strategy for treating very small vessel CAD because the data is sparse.^11^, ^12^ Therefore, our study aimed to assess the long‐term clinical outcomes of patients treated with 2.0‐mm zotarolimus DES for very small vessel CAD.

## 2 METHODS

### 2.1 Study population

This single‐arm retrospective observational study included patients with very small vessel CAD from Kaohsiung Veterans General Hospital, Kaohsiung, Taiwan, for the preriod of December 2016 to May 2020. Very small vessel CAD was defined as a lesion with RVD < 2.25 mm. The study only included patients with very small vessel CAD that are treated with 2.0‐mm zotarolimus DES and were clinically followed up for 2 years. The exclusion criteria were being older than 95 years, having advanced cancer, or being lost follow‐up after PCI. The Human Research Committee of Kaohsiung Veterans General Hospital approved the study protocol, and written informed consent was waived due to the study's retrospective nature (KSVGH22‐CT8‐02). This study was performed under relevant guidelines and regulations.

### 2.2 Interventional procedures

PCI was performed using standard procedure, and the specific revascularization technique was chosen based on the operator's judgment. All patients received a loading dose of aspirin (300 mg) and P2Y12 inhibitors (clopidogrel 300 mg for stable angina or ticagrelor 180 mg for acute myocardial infarction [AMI]) before the PCI. Unfractionated heparin (7500‐IU bolus) was administered before the intervention to achieve an activated clotting time of >300 s. When a very small vessel lesion was considered hemodynamically significant, 400−800 µg of nitroglycerine was administered intracoronary to reassess the vessel size and distinguish it from coronary spasm. Lesions were treated with 2.0‐mm zotarolimus DES if the following results were observed after predilated balloon angioplasty: flow‐limiting dissection, thrombolysis in myocardial infarction (TIMI) flow < 3, or residual stenosis > 40%. Following PCI, P2Y12 inhibitors were recommended to be continued (clopidogrel 75 mg/day or ticagrelor 90 mg twice daily) for at least 1 year and continue aspirin (100 mg/day) or P2Y12 inhibitor (if aspirin intolerant) indefinitely. Angiotensin‐converting enzyme inhibitors and beta‐blockers were prescribed for patients presenting with AMI unless contraindicated. Statin prescriptions were granted according to lipid guidelines in Taiwan and regulations of Taiwan's National Health Insurance Administration.^13^ For patients undergoing PCI for stable angina, other medications, such as beta‐blockers or nitrates, were administered at the attending physician's discretion.

### 2.3 Angiographic analysis

Angiographic data were measured using quantitative coronary angiography analysis, including RVD, minimum lumen diameter, and percent of stenosis. The complexity of the lesions was defined according to the modified American College of Cardiology/American Heart Association grading system.^14^, ^15^ This modified classification divided the coronary lesions into three categories (A, B, and C) based on 11 angiographic criteria. Within the intermediate risk category B, subcategories B1 and B2 are distinguished by the number of unfavorable characteristics present.^14^ Calcification was classified into none/mild, moderate, and severe.^16^ Moderate calcification was defined as radiopacity apparent only during the cardiac cycle before contrast injection. Severe calcification was defined as apparent radiopacity without cardiac motion, affecting both sides of the arterial lumen. Postdilation was indicated by the presence of additional dilatations induced by *a* > 2.0‐mm noncompliant balloon within the stent area following stent deployment. Lesion success was defined as residual stenosis < 30%, final TIMI flow grade of 3, and absence of significant side branch occlusion or flow‐limiting dissection after the procedure. Follow‐up coronary angiography was performed only in symptomatic patients with myocardial ischemia documented by a noninvasive test.

### 2.4 Study definitions and endpoints

The primary endpoint of the study was the occurrence of major adverse cardiovascular events (MACE) at the second year, defined as a composite of all‐cause death, nonfatal myocardial infarction (MI), and clinically‐driven target lesion revascularization (TLR). TLR was defined as any repeated revascularization procedure performed for >50% angiographic narrowing of the treated lesion from 5 mm proximal to 5 mm distal to the stent. An additional endpoint included stent thrombosis, which was classified into early (within one month), late (between 1 and 12 months), or very late (after 12 months). Deaths that could not be attributed to a clear cause were defined as being cardiac in origin. CKD was indicated if estimated glomerular filtration rate < 60 mL/min/1.73 m^2^ for >3 months and if end‐stage renal disease was present during dialysis. Patients with a history of previous MI, previous PCI, or previous coronary artery bypass graft surgery were defined as having established CAD. Data on demographic characteristics, procedures, and outcomes were collected during clinical visits or obtained from electronic medical records. Part of the data were from the Department of Medical Education and Research Center of Medical Informatics in Kaohsiung Veterans General Hospital.

### 2.5 Statistical analyses

The findings are presented as the mean and standard deviation (SD) for continuous variables and as the frequency for categorical variables. Student's *t*‐test or the Wilcoxon rank‐sum test was used to compare the baseline clinical and angiographic characteristics for continuous variables between groups. The *χ* ^2^ test or Fisher's exact test (when any expected cell count was <5 for a 2 × 2 table) were applied for categorical variables. A binary logistic model was used in a multiple regression to identify variables predicting MACE. The candidate variables were significant indicators at *p* < 0.10 in the univariate analysis. Hosmer−Lemeshow goodness‐of‐fit tests were applied. A backward variable selection approach was used for the final model. A two *p* < 0.05 indicated significance. All statistical analyses were performed using SPSS, version 24.0 (IBM).

## 3 RESULTS

### 3.1 Patient characteristics

A total of 141 patients treated with 2.0 mm DES were identified during the study period. We excluded 1 patient over 95 years old, 2 patients with advanced cancer, and 4 patients who were lost in follow‐up, leaving 134 patients for analysis. At 2‐year follow‐up, MACE was noted in 27 patients (20.1%).

The baseline demographics and clinical characteristics are summarized in Table 1. The mean age of the study population was 63.5 ± 11.7 years, and men were overrepresented (79.9%). Almost 25% of patients presented with acute coronary syndrome (22.4%). In all patients with ST‐elevation MI, the stented lesions were in the culprit vessels. The patients with MACE had a high percentage of established CAD (59.3%), DM (77.8%), and CKD (59.3%), which indicated that this population belonged to the high‐risk group.

**Table 1:** Patient demographics and baseline clinical characteristics.

	Total population (*N* = 134)	MACE (*N* = 27)	No MACE (*N* = 107)	*p* Value
Demographic findings				
Age	63.51 ± 11.715	65.59 ± 12.933	62.99 ± 11.393	0.304
Age > 75‐years	23 (17.2)	6 (22.2)	17 (15.9)	0.408
Sex (male)	107 (79.9)	23 (85.2)	84 (78.5)	0.614
BMI (kg/m^2^)	26.346 ± 3.550	25.395 ± 3.747	26.586 ± 3.475	0.208
Smoking status				0.026
Nonsmoker	57 (42.5)	6 (22.2)	51 (47.7)	
Current smoker	30 (22.4)	6 (22.2)	24 (22.4)	
Ex‐smoker	47 (35.1)	15 (55.6)	32 (29.9)	
Diabetes mellitus	79 (59.0)	21 (77.8)	58 (63.1)	0.045
Dyslipidemia	76 (56.7)	13 (48.1)	63 (58.9)	0.431
Hypertension	92 (68.7)	17 (70.1)	75 (63.0)	0.630
CKD	40 (29.9)	16 (59.3)	24 (22.4)	<0.001
Heart failure	33 (24.6)	9 (33.3)	24 (22.4)	0.355
Established CAD	55 (41.0)	16 (59.3)	39 (36.4)	0.053
Atrial fibrillation	21 (15.7)	6 (22.2)	15 (14.0)	0.373
Previous MI	19 (14.2)	7 (25.9)	12 (11.2)	0.065
Previous stroke	10 (7.5)	2 (11.1)	8 (6.5)	0.421
Presentation				0.279
Angina	104 (77.6)	19 (70.4)	83 (79.4)	
Unstable angina	1 (0.7)	0 (0.00)	1 (0.9)	
NSTEMI	23 (17.2)	5 (18.5)	18 (16.8)	
STEMI	6 (4.5)	3 (11.1)	3 (2.8)	
Clinical data				
Hemoglobin (g/dL)	12.89 ± 2.43	12.29 ± 2.65	13.04 ± 2.37	0.113
Hemoglobin < 11 g/dL	34 (25.4)	12 (44.4)	22 (20.6)	0.021
Creatinine (mg/dL)^a^	1.22 (1.00−1.60)	1.47 (1.11−6.22)	1.17 (0.98−1.46)	0.001
Triglyceride (mg/dL)	149.61 ± 135.62	139.41 ± 78.01	152.19 ± 146.80	0.863
HDL‐cholesterol (mg/dL)	43.78 ± 10.84	42.59 ± 9.70	44.08 ± 11.13	0.675
LDL‐cholesterol (mg/dL)	107.22 ± 27.43	108.30 ± 41.90	106.95 ± 22.64	0.920
HbA1c (%)	6.59 ± 1.40	6.89 ± 1.80	6.52 ± 1.28	0.770
LV ejection fraction (%)	49.60 ± 7.437	46.04 ± 8.81	50.50 ± 6.80	0.030
Medications				
Aspirin	122 (91.0)	24 (88.9)	98 (91.6)	0.707
Clopidogrel	88 (65.7)	14 (51.9)	74 (69.2)	0.143
Ticagrelor	33 (24.6)	8 (29.6)	25 (23.4)	0.671
Prasugrel	13 (9.7)	6 (22.2)	7 (6.5)	0.024
Beta‐blocker	72 (53.7)	13 (48.1)	59 (55.1)	0.663
ACEI or ARB	73 (54.5)	13 (48.1)	60 (56.1)	0.601
Statin	97 (72.4)	14 (51.9)	83 (77.6)	0.015

*Note*: Data are presented as absolute numbers and percentages or means ± standard deviation, unless otherwise specified.

Abbreviations: ACEI, angiotensin‐converting enzyme inhibitor; ARB, angiotensin‐receptor blocker; BMI, body mass index; CAD, coronary artery disease; CKD, chronic kidney disease; HDL, high density lipoprotein; LDL, low density lipoprotein; LV, left ventricular; MACE, major adverse cardiac events; MI, myocardial infarction; NSTEMI, non ST‐elevation myocardial infarction; STEMI, ST‐elevation myocardial infarction.

^a^ Creatinine (mg/dL) is not normally distributed and is presented as median (interquartile range).

### 3.2 Angiographic findings and procedure characteristics

Results on lesion characteristics and procedures are displayed in Table 2. Approximately 90% of the study sample had multivessel diseases, predominantly triple vessel disease (71.6%). The target lesions were small and diffuse, with a mean length of 20.9 ± 5.51 mm and an RVD of 1.91 ± 0.16 mm. Some calcification was observed in angiography findings (moderate/severe: 29.1%). Three patients had highly calcified lesions that required debulking by rotablator atherectomy. Most lesions were anatomically complex (type B2/C: 90.3%), and almost 25% of the stents were placed in chronic total occlusion (CTO) lesions (23.9%). Intravascular ultrasound was used for approximately 25% of the patients (25.4%), mainly for evaluations of CTO lesions. Almost all patients received predilated balloon angioplasty before stenting. (99.3%). The procedures had a low incidence of acute complications, such as stent loss or coronary perforations. The 3 cases of distal wire perforations occurred during the CTO intervention, which were successfully treated using prolonged small balloon inflations. Approximately one‐third of the patients underwent follow‐up angiography (34.3%) due to symptomatic angina or positive functional study results.

**Table 2:** Lesion and procedural characteristics.

	Total population (*N* = 134)	MACE (*N* = 27)	No MACE (*N* = 107)	*p* Value
Diseased vessels				0.110
Single vessel disease	13 (9.7)	0 (0.00)	13 (10.4)	
Double vessel disease	25 (18.7)	4 (14.8)	21 (19.6)	
Triple vessel disease	96 (71.6)	23 (19.3)	73 (76.7)	
Target coronary artery				0.649
Left anterior descending artery	53 (39.6)	13 (48.1)	40 (37.4)	
Left circumflex artery	52 (38.8)	8 (29.6)	44 (41.1)	
Right coronary artery	28 (20.9)	6 (22.2)	22 (20.6)	
Intermediate artery	1 (0.7)	0 (0.0)	1 (0.9)	
Modified ACC/AHA lesion type				0.401
A	2 (1.5)	0 (0.00)	2 (1.9)	
B1	11 (8.2)	2 (7.4)	9 (8.4)	
B2	45 (33.6)	6 (22.2)	39 (36.4)	
C	76 (56.7)	19 (70.4)	57 (53.3)	
Calcification				0.074
None or mild	95 (70.9)	15 (55.6)	80 (74.8)	
Moderate	30 (22.4)	8 (29.6)	22 (20.6)	
Severe	9 (6.7)	4 (14.8)	5 (4.7)	
Chronic total occlusion	32 (23.9)	9 (33.3)	23 (21.5)	0.300
Preprocedure measurements				
Lesion length (mm)	20.9 ± 5.51	22.56 ± 5.36	20.48 ± 5.50	0.081
Lesion length > 20 mm	68 (50.7%)	17 (63.0)	51 (47.7%)	0.228
Reference vessel diameter (mm)	1.91 ± 0.16	1.89 ± 0.17	1.92 ± 0.16	0.728
Mean % stenosis	87.90 ± 9.18	89.85 ± 7.91	87.40 ± 9.44	0.285
Predilation	133 (99.3)	27 (100.0)	106 (99.1)	1.000
Postdilation	58 (43.3)	14 (51.9)	44 (41.1)	0.431
Intravascular ultrasound	34 (25.4)	10 (37.0)	24 (22.4)	0.190
Rotablation artherectomy	3 (2.2)	1 (3.70)	2 (1.9)	0.494
IABP placement	1 (0.7)	1 (3.7)	0 (0.0)	0.201
Coronary perforation	3 (2.2)	0 (0.0)	3 (2.8)	1.000
Stent loss	0 (0.0)	0 (0.0)	0 (0.0)	
History of BMS implantation in nontarget lesion	12 (9.0)	5 (18.5)	7 (6.5)	0.065
Lesion success	137 (100.00)	27 (100.00)	107 (100.00)	
Follow‐up angiography	46 (34.3)	19 (70.4)	27 (25.2)	<0.001

*Note*: Data are presented as absolute numbers and percentages or means ± standard deviation.

Abbreviations: BMS, bare‐metal stent; IABP, intra‐aortic balloon pump; MACE, major adverse cardiac events.

### 3.3 Clinical outcomes

Table 3 depicts the clinical outcomes of the study. MACE at 1 year follow‐up occurred in 15 (11.2%) patients and 27 (20.1%) patients at the 2‐year follow‐up. The adverse events during the follow‐up period were mainly attributed to in‐stent restenosis requiring revascularization (11.9%). One case of late stent thrombosis was noted 8 months after the index procedure and resulted in a nonfatal MI. Seven patients died from noncardiac causes (Supporting Information: Material 1), and noncardiac death is another major cause of MACE.

**Table 3:** Clinical outcomes at 2‐year follow‐up; total (*N* = 134).

Clinical Outcomes, *N* (%)	At 6‐month	At 1‐year	At 2‐year
MACE	6 (4.5)	15 (11.2)	27 (20.1)
All cause death			
Cardiac death	1 (0.7)	3 (2.2)	3 (2.2)
Noncardiac death	2 (1.5)	5 (3.7)	7 (5.2)
Tartget vessel myocardial infarction	1 (0.7)	1 (0.7)	1 (0.7)
Target lesion revascularization	2 (1.5)	6 (4.5)	16 (11.9)
Stent thrombosis			
Early thrombosis	0 (0.0)	0 (0.0)	0 (0.0)
Late thrombosis	0 (0.0)	1 (0.7)	1 (0.7)
Very late thrombosis	0 (0.0)	0 (0.0)	0 (0.0)

*Note*: Data are presented as absolute numbers and percentages, unless otherwise specified.

Abbreviation: MACE, major adverse cardiac events.

To investigate the factors associated with the long‐term outcome, the MACE predictor candidates were variables that were significant at *p* < 0.10 in the univariate analysis; these were DM, CKD, heart failure, anemia (Hgb < 11 g/dL), established CAD, calcification (moderate and severe degree), and statin use. Results from the multiple logistic regression indicated that the presence of CKD (odds ratios [OR]: 4.291; 95% confidence interval [CI]: 1.574−11.704, *p* = 0.004) and moderate to severe degrees of calcification (OR: 3.688; 95% CI: 1.311−10.371, *p* = 0.013) were independent predictors of worse long‐term outcomes (Table 4). Statin use was an indicator of a lower likelihood of adverse events (OR: 0.335; 95% CI: 0.119−0.949, *p* = 0.040). Hosmer‐Lemeshow tests validated our risk prediction models (*p* = 0.857).

**Table 4:** Predictors of MACE by univariable and multiple logistic regression analysis.

	Univariable model			Multiple model		
	OR	95% CI	*p* Value	OR	95% CI	*p* Value
Age > 75‐years	1.625	0.568−4.650	0.365	－	－	－
Male gender	1.574	0.495−5.010	0.442	－	－	－
Current smoker	0.988	0.358−2.726	0.982	－	－	－
Diabetes mellitus	2.957	1.106−7.908	0.031	－	－	－
Dyslipidemia	0.649	0.278−1.513	0.317	－	－	－
Hypertension	0.725	0.300−1.756	0.476	－	－	－
CKD	5.030	2.062−12.274	<0.001	4.291	1.574−11.704	0.004
Heart failure	3.409	1.425−8.154	0.006	－	－	－
Established CAD	2.536	1.070−6.010	0.034	2.410	0.923−6.293	0.072
Atrial fibrillation	1.752	0.608−5.052	0.299	－	－	－
Previous stroke	1.786	0.430−7.417	0.425	－	－	－
Hb < 11 g/dL	3.091	1.267−7.543	0.013	－	－	－
Calcification^a^	2.370	0.988−5.689	0.053	3.688	1.311−10.371	0.013
Tortuosity	0.633	0.133−3.015	0.566	－	－	－
Lesion length > 20 mm	1.867	0.783−4.448	0.159			
Chronic total occlusion	1.826	0.725−4.599	0.201			
Intravascular ultrasound	2.034	0.824−5.022	0.123	－	－	－
Aspirin use	0.735	0.185−2.923	0.662	－	－	－
P2Y12 inhibitors use^a^	0.560	0.135−2.326	0.425	－	－	－
Beta‐blocker use	0.755	0.324−1.760	0.516	－	－	－
Statin use	0.311	0.129−0.752	0.009	0.335	0.119−0.949	0.040
ACEI or ARB use	0.727	0.312−1.695	0.461	－	－	－

*Note*: *P2Y12 inhibitors use is defined as either prescription of Clopidogrel, Ticagrelor, or Prasugrel.

Abbreviations: ACEI, angiotensin‐converting enzyme inhibitor; ARB, angiotensin‐receptor blocker; CAD, coronary artery disease; CKD, chronic kidney disease; MACE, major adverse cardiac events; OR, odds ratio; 95% CI, Confidence interval.

^a^ Calcification is defined as the combination of moderate and severe calcifications.

## 4 DISCUSSION

Findings on very small vessel CAD interventions (RVD: 2.0−2.25 mm) remain limited, and the long‐term performance of 2.0‐mm zotarolimus DES in real‐world practice is unclear. Our contribution is our results on long‐term outcomes for a high‐risk, very small vessel CAD cohort undergoing stenting. The analysis found that 2.0‐mm zotarolimus DES yielded favorable short‐term and medium‐term results for complex lesions; however, increased rates of MACE were observed at 2 years. CKD and calcified lesions were independent risk factors for MACE after PCI with 2.0‐mm zotarolimus DES, whereas statin was associated with improved outcomes. The incidence of stent thrombosis was low throughout the follow‐up period, and the adverse events were mainly driven by late lumen loss.

In the prospective study of Price et al., treatment with 2.0‐mm zotarolimus DES resulted in low rates of MACE (5.0%) and TLR (2.0%) and no incidence of stent thrombosis at 12 months.^10^ The higher event rates in our cohort could be partly explained by its all‐comers population. With multiple comorbidities and higher proportions of AMI on presentation (our study: 21.7% vs. Price et al.: 1.0%). Our study's higher rate of noncardiac death also suggests that this cohort included more fragile subsets with CAD.^17^ Furthermore, approximately 40% of our study population had established CAD, a reported risk factor for worse outcomes after PCI,^10^ and they markedly differed from patients with de novo lesions in a previous trial.^17^ Furthermore, the coronary lesions in our study were composed of more difficult subsets, including type B2/C lesions, (our study: 90.3% vs. Price et al.: 65.4%), calcifications (our study: 29.1% vs. Price et al.: 24%), CTO lesions (our study: 25.9% vs. Price et al.: not reported), and longer lesion length (our study: 20.9 mm vs. mean Price et al.: 12.6 mm).^10^, ^18^, ^19^, ^20^ Because the very small vessel lesions are commonly found in patients with DM or CKD and are manifested with diffuse lesions in nature, our study might better reflect the outcome of very small vessel stenting in routine clinical settings.^21^, ^22^

Stent delivery is technically difficult in coronary interventions for smaller vessel sizes because manipulation of the stent is usually hampered by calcification, vessel tortuosity, and the distal location of the lesion.^23^ Dislodgment of 2.0‐mm DES was reported in a study comparing different strategies for treating CAD with very small vessel sizes.^24^ The same study also found a higher incidence of stent thrombosis at 1 year (2%), which could be attributed to malposition or stent underexpansion.^24^ However, the findings from other studies and our study indicate that 2.0‐mm zotarolimus DES provides appropriate lesion crossability and a low rate of stent thrombosis (≤1%).^10^, ^25^ In these studies, almost all very small vessel lesions were predilated and approximately 40% of the implanted stents were postdilated.^10^, ^25^ These findings highlight the importance of thorough preparation before stenting and of stent expansion in very small vessel interventions. Our study indicates that calcification is still an independent predictor of a worse prognosis despite the feasibility of 2.0‐mm DES for a calcified lesion. The effect of calcification on outcomes following PCI appeared to be higher in very small vessel stents (OR: 3.69, 95% CI: 1.31−10.37) than in the large stents used in other studies (HR: 1.18, 95% CI: 1.01−1.39).^26^ The very small vessel diameter poses an obstacle to intravascular imaging, cutting or scoring balloons, and debulking devices and might be associated with the suboptimal results in calcified lesion preparation.

Although stenting has the advantage of acute lumen gain, long‐term efficacy is *a major* concern. Studies have indicated that a vessel diameter < 2.50 mm could serve as a threshold for small target vessels to predict higher rates of 2‐year MACE (10.8%) and restenosis (4.8%), and studies have found no significant association between outcomes and vessel diameter.^5^ Another pooled analysis reported no significant difference in long‐term outcomes between vessel diameters of ≤2.25 and >2.25−2.75 mm (MACE: 15.4% vs. 11.5%, HR: 1.3, 95% CI: 1.0−1.7, *p* = 0.12; TLR: 6.9% vs. 4.5%, HR: 1.4, 95% CI: 1.0−1.7, *p* = 0.17).^9^ However, target vessel revascularization occurred more frequently in patients with vessel diameter ≤ 2.25 mm (11.2% vs. 7.6%, HR: 1.5, 95% CI: 1.1−2.1, *p* = 0.02). Consistent with our findings of an RVD of nearly 2.0 mm (MACE: 20.1% and TLR: 11.9%), worse prognosis was noted for stents in very small vessels. Stent‐related neointimal hyperplasia is more critical in a 2.0‐mm‐diameter vessel than in a larger vessel because smaller vessels are less able to accommodate neointimal tissue without compromising blood flow. Our study indicated that late lumen loss still deserves the attention of PCI in these very small vessels. Future prospective studies should compare strategies, such as drug coated balloon or conventional medical treatment, to provide clinical insight into very small vessel lesions.^7^, ^24^

Our study revealed that noncardiac death is the primary cause of mortality after stenting in very small vessel CAD. This finding is consistent with the device's safety, PCI technique advances, and secondary preventative therapies' effectiveness. However, it also suggested that most of our patients have noncardiac comorbidities at the time of PCI. This result should be considered during patient selection, especially in those with multiple noncardiac comorbidities, poor functional status, or less certain indications for PCI, since an aggressive revascularization strategy could make these patients even more complex (e.g., contrast‐induced acute kidney injury or gastrointestinal bleeding after dual antiplatelet therapies).

CKD and higher cardiovascular event rates following PCI have been well documented.^27^, ^28^, ^29^ Our research demonstrates that CKD is a strong risk factor for worse prognosis after very small vessel intervention (OR: 4.291, 95% CI: 1.574–11.704, *p* = 0.004). The decision between revascularization and conventional medical treatment is more serious for patients with CKD with very small vessel lesions. Obstructive lesions with smaller RVD can still affect the coronary blood flow and myocardial perfusion and result in angina, impaired quality of life, and ventricular arrhythmias.^30^ Some studies have also indicated that complete revascularization in patients with CKD may attenuate the risk of sudden death or lethal arrhythmia by increasing the myocyte reserve to handle fluxes in fluid and electrolytes or transient changes in sympathetic tone.^31^, ^32^, ^33^ However, PCI of very small vessels may expose these patients to acute procedural complications, increase their risk of bleeding from dual‐antiplatelet therapy, and subsequent restenosis requiring repeated angioplasty.^34^, ^35^, ^36^ In general, treatment strategies should be individualized for this high‐risk population of patients with CKD.

Previous trials have reported that statin therapy is associated with improved survival and reduced restenosis after coronary stent placement.^37^, ^38^, ^39^ A large cohort study found that Asian patients after PCI would benefit from high‐intensity statins for long‐term MACE, irrespective of whether they have attained their LDL‐cholesterol target.^40^ The efficacy of statins appears to be independent of their lipid‐lowering effects. The result of our study supported the fidnings that statin therapy was associated with improved clinical outcomes after PCI in very small vessel lesions (OR: 0.335, 95% CI: 0.119−0.949, *p* = 0.040). Small vessel coronary artery stenosis is frequently found in patients with DM and CKD and who smoke and is characterized by chronic low‐grade inflammation.^1^, ^41^ The vascular injury from balloon angioplasty and stent implantation would also lead to platelet activation and inflammation within the vessel wall and the distal microvasculature.^42^, ^43^ Therefore, the pleiotropic effects of statin on anti‐inflammation, anti‐proliferation, and anti‐thrombosis may play a more significant role in very small vessel interventions. A statin prescription should be considered regardless of dyslipidemia for patients who have undergone coronary stenting in vessels with a very small diameter.

This study has the following limitations. First, the retrospective study design, lack of randomization, lack of control group, and limited sample size made it impossible to control for all confounders and might have led to inherent selection bias. The patients' background parameters also varied to a certain extent, and there were no planned sensitivity analyses to assess the findings of the primary analysis. Therefore, the risk model identified in our study should still be interpreted with caution due to the width of 95% CI and the potential effects of other undetected variables. Second, routine follow‐up coronary angiography was not performed because of cost and ethical considerations. The potential in‐stent late loss was not comprehensively assessed. Third, although our hospital is a tertiary medical center and disease management accords with current standards of clinical practice, the result of this single‐center study may not be generally applicable to other health facilities.

## 5 CONCLUSION

Our study reports that 2.0‐mm zotarolimus DES for very small vessel CAD is feasible on the basis of findings obtained at 2‐year follow‐up. Statin therapy was associated with fewer incidences of MACE. CKD and calcified lesions were independent predictors of long‐term MACE rate in patients treated with 2.0‐mm zotarolimus DES. The decision to stent very small coronary vessels in these high‐risk patients should be tailored to each patient's unique clinical characteristics, and clinicians should ensure adequate lesion preparation and optimal stent deployment if revascularization by PCI is required.

## CONFLICT OF INTEREST STATEMENT

The authors declare no conflict of interest.

## Supporting information

Supporting information.

## Data Availability

The data that support the findings of this study are available on request from the corresponding author. The data are not publicly available due to legal restrictions imposed by the government of Taiwan in relation to the “Personal Information Protection Act.”
