# Supplementary material for: Long‐term outcomes after stent implantation in very small vessel coronary artery disease
Source: Clin Cardiol. 2023 Feb 23;46(4):431–40. doi: 10.1002/clc.24000 (PMC10106663; doi:10.1002/clc.24000)
Supplement: Supplementary file 1 — Supporting information. [file CLC-46-431-s001.docx]

Supplementary Table 1. Causes of non-cardiac deaths

|  | **Presentation** | **Time from coronary intervention to event** | **Medical history** | **Cause of non-cardiac death** |
| --- | --- | --- | --- | --- |
| 78-year-old male | Non-ST-elevation myocardial infarction | 247 days | 1. Coronary artery disease, triple vessel disease 2. Heart failure with reduced ejection fraction 3. Peripheral arterial occlusion disease status post amputations of right 2nd and 3rd toes 4. Type 2 diabetes mellitus 5. End-stage renal disease on dialysis 6. Secondary hyperparathyroidism status post parathyroidectomy | Community-acquired pneumonia, bilateral lungs, with acute respiratory failure and  septic shock |
| 69-year-old male | Stable angina  (Reversible defect  on thallium scan) | 454 days | 1. Coronary artery disease, left main and triple vessel disease 2. Heart failure with reduced ejection fraction 3. Sick sinus syndrome status post permanent pacemaker 4. Old ischemic stroke 5. Type 2 diabetes mellitus 6. Dyslipidemia 7. End-stage renal disease on dialysis | Infected pressure ulcers with bacteremia, respiratory failure and septic shock |
| 84-year-old female | Stable angina (Reversible defect  on thallium scan) | 243 days | 1. Coronary artery disease, triple vessel disease 2. Heart failure with preserved ejection fraction 3. Atrial fibrillation 4. Peripheral arterial occlusion disease 5. Hypertension 6. Type 2 diabetes mellitus 7. End-stage renal disease on dialysis | Pneumonia, bilateral lungs, with Escherichia coli bacteremia and septic shock |
| 50-year-old male | Recent myocardial infarction with heart failure and angina | 83 days | 1. Coronary artery disease, left main and triple vessel disease 2. Heart failure with reduced ejection fraction 3. Myocardial infarction 4. Type 2 diabetes mellitus | Necrotizing fasciitis of left lower limb,  with abscess formation and septic shock |
| 77-year-old female | Stable angina (Reversible defect  on thallium scan) | 47 days | 1. Coronary artery disease, left main and triple vessel disease 2. Hypertension 3. Type 2 diabetes mellitus 4. End-stage renal disease on dialysis | Hospital-acquired pneumonia, with acute respiratory failure and septic shock |
| 75-year-old male | Stable angina  and intradialytic hypotension | 703 days | 1. Coronary artery disease, triple vessel disease 2. Peripheral arterial occlusion disease with chronic limb ischemia 3. Hypertension 4. Dyslipidemia 5. Type 2 diabetes mellitus 6. End-stage renal disease on dialysis 7. Gastric ulcers | Hyperkalemia, end-stage renal disease and gastrointestinal bleeding related |
| 74-year-old male | ST elevation myocardial infarction | 348 days | 1. Coronary artery disease, triple vessel disease 2. Hypertension 3. Type 2 diabetes mellitus 4. Liver cirrhosis, hepatitis B virus related,  Child-Pugh class A 5. Hepatocellular carcinoma, pT1N0M0, stage I, status post segementectomy of S5 and S6 (10 years before coronary intervention) | Hospital-acquired pneumonia, bilateral lungs, Klebsiella pneumoniae related, with septic shock and disseminated intravascular coagulopathy |
